# Establishing Australia’s national linked COVID Register

**DOI:** 10.23889/ijpds.v9i5.2805

**Published:** 2024-09-18

**Authors:** Louise Gates, Claudia Slimings, Bronte O’Donnell, Anna Reynolds, Claire Sparke, Vivien Luong, Daniel Palamara, Renee Foo, Mariam Bakshi, Vanessa D'Souza

**Affiliations:** 1 Australian Institute of Health and Welfare

## Objectives and Approach

The Australian Institute of Health and Welfare (AIHW) is an independent statutory Australian Government agency tasked with collating health data across federal and state/territory governments, and is accredited to provide complex integration, de-identification and secure access to linked data.

To support research into the COVID pandemic, the AIHW has created a national-level COVID Register, which integrates COVID case data from infectious disease registers, and data from hospitals and intensive care, immunisation, deaths, health system usage data, aged care and disability support.

## Results

Data were probabilistically linked using the AIHW’s linkage spine, matching names, addresses, date of birth and sex. The AIHW data linkage protocols are based on the Five Safes framework which reinforce management of the privacy and confidentiality of data.

Linkage rates for COVID cases across participating jurisdictions generally exceeded 95%. Ongoing work aims to increase the coverage of COVID cases, both in time and to include case data from one remaining jurisdiction.

## Conclusions

Linking disparate datasets from multiple sources has created a critical resource for exploring COVID and patient journeys in Australia. Currently, the Register supports projects to estimate expenditure associated with COVID, describe the impact of COVID for subgroups such as people living rural and remote areas, or those with dementia.

## Implications

Ultimately, the Register will be integrated into the National Health Data Hub to enable ongoing health research and analysis through enduring national linked health data. The value of the Register extends beyond COVID and into other infectious diseases and chronic conditions.

